# Sales and pricing decisions for HIV self-test kits among local drug shops in Tanzania: a prospective cohort study

**DOI:** 10.1186/s12913-021-06432-1

**Published:** 2021-05-06

**Authors:** Calvin Chiu, Lauren A. Hunter, Sandra I. McCoy, Rashid Mfaume, Prosper Njau, Jenny X. Liu

**Affiliations:** 1 School of Public Health, University of California, 2121 Berkeley Way, Berkeley, 94704 CA USA; 2 Shinyanga Regional Medical Office, Shinyanga, Tanzania; 3 Health for a Prosperous Nation, Dar es Salaam, Tanzania; 4 National AIDS Control Programme, Ministry of Health, Community Development, Gender, Elderly, and Children, Dar es Salaam, Tanzania; 5 Institute for Health and Aging; Bixby Center for Global Reproductive Health, University of California, San Francisco, CA USA

**Keywords:** HIV self-testing, Drug shops, Pricing, Private sector distribution

## Abstract

**Background:**

Public health initiatives must look for ways to cost-effectively scale critical interventions to achieve high coverage. Private sector distribution channels, can potentially distribute preventive healthcare products to hard-to-reach populations, decongest public healthcare systems, and increase the sustainability of programs by getting customers to share costs. However, little is known about how sellers set prices for new products. By introducing a new product, HIV self-test kits, to local drug shops, we observed whether shops experimented with pricing, charged different buyers different prices, and whether prices converged within the local market over our study period.

**Methods:**

From August to December 2019, we provided free HIV self-test kits, a new product, to 26 drug shops in Shinyanga, Tanzania to sell to the local community. We measured sales volume, price, customer age and sex using shop records. Using a multiple linear regression model, we conducted F-tests to determine whether shop, age, sex, and time (week) respectively were associated with price. We measured willingness-to-pay to restock test kits at the end of the study.

**Results:**

514 test kits were sold over 18 weeks; 69% of buyers were male, 40% were aged 25–34 and 32% aged 35–44. Purchase prices ranged from 1000 to 6000 Tsh (median 3000 Tsh; ~$1.30 USD). Within shops, prices were 11.3% higher for 25–34 and 12.7% higher for 45+ year olds relative to 15–19-year olds (*p* = 0.029) and 13.5% lower for men (*p* = 0.023) on average. Although prices varied between shops, prices varied little within shops over time, and did not converge over the study period or cluster geospatially. Mean maximum willingness-to-pay to restock was 2000 Tsh per kit.

**Conclusions:**

Shopkeepers charged buyers different prices depending on buyers’ age and sex. There was limited variation in prices within shops over time and low demand among shopkeepers to restock at the end of the study. Given the subsidized global wholesale price ($2 USD or ~ 4600 Tsh), further demand creation and/or cost-reduction is required before HIV self-test kits can become commercially viable in drug shops in this setting. Careful consideration is needed to align the motivations of retailers with public health priorities while meeting their private for-profit needs.

**Supplementary Information:**

The online version contains supplementary material available at 10.1186/s12913-021-06432-1.

## Background

Private sector distribution channels, such as drug shops, are underutilized when distributing preventive healthcare products, like HIV self-testing, to hard-to-reach populations [1–5]. Drug shops are critical in healthcare provision in low- and middle-income countries [6, 7], where they are often the first point of access for drugs, contraceptives, pregnancy tests, and informal counseling and referral [8–12]. Drug shops are an attractive alternative to conventional facility-based HIV counselling and testing. Customers can avoid long waits and potentially rude clinic staff [13, 14], access services more discreetly with potentially less stigma, and purchase complementary products with a one-stop shop experience. Given the potential to increase community-based access, governments and donors exploring distribution strategies for HIV self-testing are strongly considering retail distribution [15]. This is potentially more sustainable than long-term free distribution since testers share costs (by paying out of pocket).

However, there is limited evidence for the commercial viability of HIV self-testing, which requires both sufficient consumer demand and interest from the retailers. An emerging body of literature estimating consumer demand for HIV self-testing [15–19] has found substantial demand at non-zero prices. Most prominently, only two studies estimate willingness-to-pay using incentive compatible elicitation methods.^1^ These studies find that consumer demand for self-testing is highly sensitive to price [19, 20], similar to evidence from other preventive healthcare technologies more generally [21]. Few studies have gauged interest from retail sellers themselves, such as drug shops, perhaps assuming that they will be willing to sell as long as there is sufficient consumer demand.

In practice, in addition to consumer demand, drug shops may consider their profit margins, cash flow, and the decisions of other competing stores when deciding to stock self-test kits and setting their sales price. This assessment is particularly difficult when introducing new products for which a strong understanding about consumer demand is lacking, and thus shops can be reluctant to take risks in stocking. This is further complicated by many drug shops’ tendency to price discriminate by selling the same product at different prices to different customers based on the seller’s perception of the customer’s willingness-to-pay [6]. Thus, understanding how drug shops make stocking and pricing decisions is critical for policymakers when considering the commercial viability of HIV self-testing, the optimal level of subsidies (if any), and which groups to target subsidies towards. These considerations apply to other preventive healthcare products in HIV, sexual reproductive health, and other public health products more generally.

We present the first systematic evidence on how drug shops set prices for HIV self-test kits by providing 26 drug shops in Shinyanga, Tanzania with free samples and observing their sales and pricing over an 18-week study period. Using data from baseline and endline shopkeeper surveys and triangulated shop and program records, we assessed (1) the variation in reported sales prices across shop, time, and observable customer characteristics, and (2) estimated willingness-to-pay to restock at the end of the study after shopkeepers had time to learn about market demand and potential competitors’ behaviors.

## Methods

### Study population

Using a prospective cohort design, we collected data from 26 drug shops (23 Accredited Drug Dispensing Outlets [ADDOs] and 3 pharmacies) in Shinyanga, a semi-urban small municipality in Tanzania, between August 2019 and December 2019.^2^ This focus on pricing behavior for new products was an ancillary study to a randomized controlled trial evaluating the impact of an intervention promoting adolescent girls and young women (AGYW) friendly services on drug shop patronage and uptake of various sexual and reproductive health services, including HIV self-testing (NCT 04045912, hereafter ‘parent study’). Twenty ADDOs were randomly sampled from a government registry; three additional ADDOs and three pharmacies were purposefully sampled to increase heterogeneity in shop size and type. Research assistants contacted the drug shop owner by phone to provide information about the study and, if interested, arrange to meet to obtain written informed consent. All participants (shopkeepers) provided written informed consent. Shops in the treatment arm were trained on contraceptive counseling for AGYW and implemented a loyalty program through which AGYW could earn prizes, discreetly request free sexual and reproductive health (SRH) products, and view an SRH product display and tablet with information videos. Shops in both treatment and control arms received training on HIV self-testing and provided self-test kits to AGYW for free. Trial details are discussed elsewhere [22]; this paper presents an ancillary study of shopkeeper behavior. Ethical approvals were obtained from the Tanzanian National Institute of Medical Research and the University of California, San Francisco.

Oral-fluid HIV self-test kits (OraQuick) were approved in Tanzania in November 2019 and not widely available during the study period. Separate from the AGYW intervention (parent study), we invited all drug shops to sell up to 60 self-test kits (given to shops for free) to the general population over the study period for whatever amount they wanted; we deliberately refrained from giving price examples during training to minimize the risk of shops being unintentionally influenced. During the study period, we neither actively encouraged nor discouraged shopkeepers and staff from communicating with each other. At the end of the study, shops were allowed to keep any unsold self-test kits to minimize any undue influence of having a study-imposed deadline on pricing or selling behavior.

### Data

First, we administered a baseline survey measuring shop and shopkeeper characteristics, including self-reported sales prices and markups of related products, such as oral contraception, emergency contraception, and pregnancy tests. All prices are measured and presented in 2019 Tanzanian shillings (Tsh) (approximately $1 USD to 2300 Tsh during the study period). Profits are defined as the difference between sales price and the restocking cost per unit. Markup (%) is defined as the difference between the sale and procurement price, as a percentage of the procurement price.

Second, shopkeepers kept standardized self-test kit sales records over the study period, which included the date, quantity, and price of each unit sold, and the age (in broad categories as perceived by the seller) and sex of the customer. To strengthen confidence in the accuracy of sales records, shopkeepers’ sales records were cross-checked against both anonymous customer feedback forms and weekly stock audits from the study team. Customers purchasing self-test kits were encouraged to fill out anonymous feedback forms with their age, sex, price paid, quantity purchased, and transaction date. Although not all buyers completed feedback forms, these data provided a lower-bound on the number of kits sold. Stock audits by the study team provided accurate data on sales between each audit, allowing us to quantify the level of missing data in the sales records. Together, these three data sources provided a robust picture of sales and pricing of self-test kits over the study period.

At the end of the study, we administered an endline survey asking shopkeepers to report their sales volume and prices in the last week and month, and beliefs about other shops’ sales volume and pricing in the last week and month. We asked them to predict hypothetical demand for self-test kits in the future (next week) at different price points: 0 (free), 2000, 5000 and 10,000 Tsh. When administering the survey, we randomized the order in which price levels were asked (ascending or descending) to minimize related response biases.

Lastly, at the end of the study, we elicited shopkeepers’ willingness-to-pay to restock self-test kits for future sale using an incentive-compatible multiple price list method [23]. We provided shops with an unexpected one-time opportunity to restock 5 kits at 6 different price levels: from 1000 to 6000 Tsh per kit in 1000 Tsh increments. We identified the maximum willingness-to-pay as the point when shopkeepers switched from answering “yes” to “no.” The research assistant rolled a die and the respondent had to purchase the additional kits at the corresponding price level if it did not exceed her reported maximum willingness-to-pay. For example, if a restocking price of 3000 Tsh was randomly selected (by rolling a ‘3’), the shopkeeper would have to purchase the 5 additional kits if their maximum willingness-to-pay price was 3000 Tsh or higher. In this way, the respondent is incentivized to truthfully report her willingness-to-pay. During the survey, respondents practiced this elicitation method to verify their understanding before being asked the actual survey question. This method to estimate demand is superior to those using stated preferences in a hypothetical scenario without real trade-offs, which often overestimates demand [19].

### Analysis

Given that the HIV self-test kit was a new product with no pre-determined market price or consumer demand information, we hypothesized that shops would experiment with pricing over time and that the sales prices for self-test kits would converge towards the end of the study period. First, we calculated descriptive statistics and tested for differences by treatment status (from the parent study) using t-tests for continuous variables and chi-squared tests for categorical variables. We then constructed scatter plots of sales prices over the study period separately by shop to look for patterns in price variation and/or convergence over time across shops, a box plot looking at sales by shop over time, and a line graph examining mean price over time across shops.

Using five different linear regression models on log price, we examined the association between price and age, sex, shop, and time (weeks since baseline) respectively. Our main outcome of interest in this analysis was the sales price of self-test kits. Sales price is logged to account for the skewed distribution and conform to normality assumptions for linear regression, as is standard practice in economic analyses of prices. All models controlled for the shop’s treatment status in the parent study’s randomized trial, and standard errors were clustered at the shop level, the unit of randomization for the parent study. First, we modelled each characteristic separately and then together with age-sex interactions to examine potential differences across subgroups along both of these dimensions (e.g. older men vs younger women). We conducted F-tests to determine the joint significance of each set of characteristics. Second, we add additional controls for shop and time fixed effects to sufficiently control for price variation between shops and across time after conducting a series of specification tests (see Additional file 1: Table S5). Model estimates were sensitive to assumptions about the correlation structure of the data, and results of Likelihood Ratio and Hausman specification tests showed that estimates with shop and time fixed effects yielded more consistent results compared to models with different random effects assumptions. We repeated the same set of models using data from the customer feedback forms as a robustness check.

For evidence of shops experimenting with prices, we estimated linear regression models looking at the association between sales price and lagged (one week) sales volume with treatment status, shop, and time fixed effects, respectively. We mapped the price of related products, mean price of self-test kits, and beliefs about other shops’ average prices to examine any geospatial clustering. We compared the degree of price variation for self-test kits, a new product, with those of existing products for which shops have had a longer time to establish pricing strategies for. We constructed box plots to assess the accuracy of shop shopkeepers’ beliefs of other shops’ sales volumes and pricing.

To estimate a “demand curve” for restocking self-test kits, we plotted the number of shops willing to restock at different price levels. We estimated seven different linear regression models to estimate the association between maximum willingness-to-pay to restock per self-test kit and various sales volume and price measures. All analysis was done using Stata version 13 (College Station, TX, USA; StataCorp LP).

## Results

Table 1 presents descriptive statistics on shop characteristics, sales volumes and prices of self-test kits, beliefs about future demand, and maximum willingness-to-pay at the end of the study. Overall, 514 self-test kits were sold by 23 shops over 18 weeks; 3 shops did not record a single sale. Sale volumes were low overall and skewed to the right (Additional file 1: Fig. S1): shops sold a median of 19 self-test kits per shop over the 18-week study period and 0 self-test kits per shop per week. Most customers were male (71%), and 40% were aged 25–34. Purchase price per self-test kit varied from 1000 to 6000 Tsh (median 3000 Tsh). About 41% of self-test kits were sold at 5000 Tsh; 26% of self-test kits were sold at 2000 Tsh.^3^ At study end, shopkeepers believed they could sell 10 and 4 self-test kits a week if priced at 2000 and 5000 Tsh, respectively, and had a median maximum willingness-to-pay to restock of 2000 Tsh per self-test kit. From the multiple price list elicitation method, 24% of shopkeepers restocked self-test kits.

**Table 1:** Shop characteristics and HIV self-test kit sales and pricing at 26 drug shops in Tanzania.

	***n*** (%)	Median (IQR)	Range	***N***
***Shop characteristics (N = 26)***				**26**
Location				
- Urban	20 (77%)			
- Peri-Urban	6 (23%)			
Years in business				
- 1 year or less	5 (19%)			
- 1–5 years	4 (15%)			
- 5–10 years	3 (12%)			
- More than 10 years	14 (54%)			
Number of employees (including owner)		1 (1)	1–4	26
***HIV self-test kit sales (N = 514)***				**514**
Per week (18 weeks)		26.5 (10)	17–50	18
Per shop^†^ (23 shops)		19 (21)	2–60	23
Per shop per week		0 (2)	0–16	437
Sex of customer				500
- Female	143 (29%)			
- Male	357 (71%)			
Age of customer				507
- 15–19	18 (4%)			
- 20–24	73 (14%)			
- 25–34	205 (40%)			
- 35–44	160 (32%)			
- 45+	51 (10%)			
***Price per kit (Tsh) (1USD ~ 2300 Tsh)***				
Over the study period		3000 (3000)	1000–6000	513
Number of test kits sold at *n*(%)				
- 1000 Tsh	36 (7%)			
- 2000 Tsh	133 (26%)			
- 2500 Tsh	25 (5%)			
- 3000 Tsh	67 (13%)			
- 4000 Tsh	40 (8%)			
- 5000 Tsh	211 (41%)			
- 6000 Tsh	1 (0%)			
Mean price per shop (23 shops)		3500 (2619)	1488–5000	23
Median price per shop (23 shops)		3000 (3000)	1000–5000	23
By sex of customer				
- Male		3000 (3000)	1000–6000	357
- Female		4000 (3000)	1000–5000	142
By age of customer				
- 15–19		1000 (1000)	1000–3000	18
- 20–24		3000 (3000)	1000–5000	73
- 25–34		3000 (3000)	1000–6000	204
- 35–44		4000 (3000)	1000–5000	160
- 45+		5000 (1000)	2000–5000	51
***Price of existing related products (Tsh)***				
Emergency contraception		5000 (0)	3000–5000	15
Oral contraception		1500 (500)	1000–2000	20
Pregnancy test		1000 (0)	600–1000	25
***Beliefs about future demand (N = 25)***				
Predicted sales next week if sold at				25
- 0 Tsh (Free)		20 (10)	3–50	25
- 2000 Tsh		10 (10)	2–30	25
- 5000 Tsh		4 (3)	0–12	25
- 10,000 Tsh		0 (1)	0–5	25
Maximum willingness-to-pay to restock		2000 (2000)	0–4000	25
Number of shops that restocked	6 (24%)			25

IQR = Inter-quartile range.^†^ Among shops that recorded at least one sale. 3 shops did not record a single sale over the study period. Rows where N does not align with the denominator (e.g., customer age and sex) for the level of observation (shop, test kit, week, etc.) reflect observations where data from some variables is missing

**Fig. 1:**
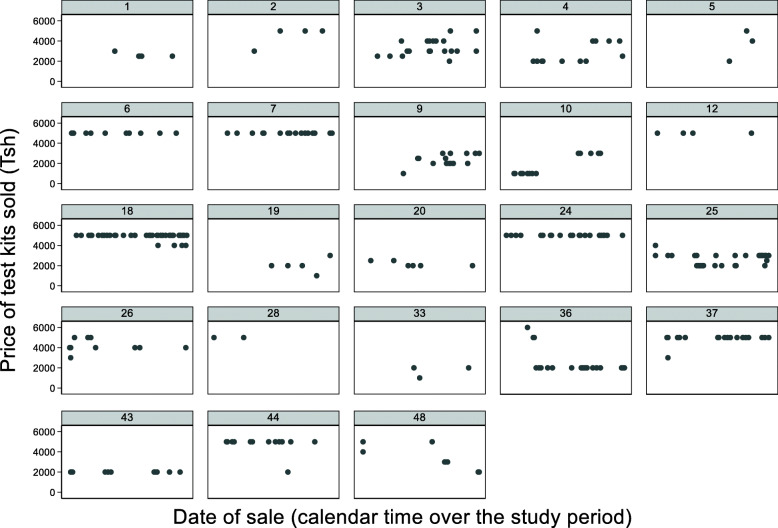
Distribution of HIV self-test kit sales price within drug shops over time. *Each graph represents a shop in our study (n = 23). Each graph plots the sales price (Tsh) of each test kit sold over the study period (August to December 2019). Blank spaces indicate no test kits sold. $1 USD approx. 2300 Tsh*

Within most shops, sales prices varied little over time (Fig. 1), with little evidence of prices converging toward a singular market price (Additional file 1: Fig. S2) despite shops initially selling self-test kits at different price levels. In contrast, reported sales prices of related products (contraceptives and pregnancy tests) varied within a narrow interval (Table 1). Similarly, the sales price of self-test kits and beliefs about other shops’ prices for self-test kits did not cluster geospatially unlike prices of related products (Additional file 1: Fig. S3).

Table 2 shows the results of five different linear regression models on the association between sales price and customer age and sex, shop fixed effects, and time. Customer age (column 1) strongly predicted prices, but customer sex did not (column 2). Across all specifications, customer age was positively correlated with prices (columns 1, 3, 4, 5). Prices were lower for men in models that included age-sex interactions (columns 3, 5); after controlling for shop and time fixed effects and including age-sex interactions (column 5), prices were 11.3% higher for 25–34 and 12.7% higher for 45+ year olds relative to 15–19 year olds (*p* = 0.029), and 13.5% lower for men relative to women (*p* = 0.023).^4^ Similar patterns held when repeated with data from the 216 customer feedback forms (Additional file 1: Table S2). The full results including iterative specification testing are shown in the Additional file 1: Table S5. Prices were not associated with sales volume once we included shop or time fixed effects (Additional file 1: Table S3).

**Table 2:** Association between HIV self-test kit sales price and observed customer characteristics

Linear regression models of log price of test kits sold (Coefficient, standard error)					
Model	1	2	3	4	5
**Predictors**	**Age**	**Sex**	**Age & Sex**	**All**	**All (age-sex)**
**Customer age**		–			
- 15–19	ref	–	ref	ref	ref
- 20–24	0.617** (0.168)	–	0.367* (0.151)	0.150** (0.044)	0.082 (0.046)
- 25–34	0.817*** (0.193)	–	0.252 (0.133)	0.231** (0.062)	0.107** (0.037)
- 35–44	0.884*** (0.207)	–	0.338* (0.151)	0.198** (0.056)	0.065 (0.057)
- 45–54	1.069*** (0.204)	–	0.535** (0.157)	0.219* (0.086)	0.120* (0.054)
**Customer sex** (Male)	–	−0.049 (0.075)	−0.608** (0.202)	−0.002 (0.026)	−0.145* (0.059)
***P*-values from F-tests of joint significance**					
Age	< 0.001	–	0.031	0.013	0.029
Sex	–	0.518	0.007	0.927	0.023
Age x Sex interaction	–	–	0.079	–	0.233
Shop	–	–	–	< 0.001	< 0.001
Week	–	–	–	0.023	0.042
**Control variables included in model**					
- Treatment status	X	X	X	X	X
- Shop fixed effects	–	–	–	X	X
- Week fixed effects	–	–	–	X	X
*R*^2^	0.166	0.0147	0.195	0.802	0.803
N	506	499	492	492	492

* *p* < 0.05, **p < 0.01, ***p < 0.001. Standard errors are clustered at the shop level. Only coefficients for customer age and sex are reported for brevity

**Fig. 2:**
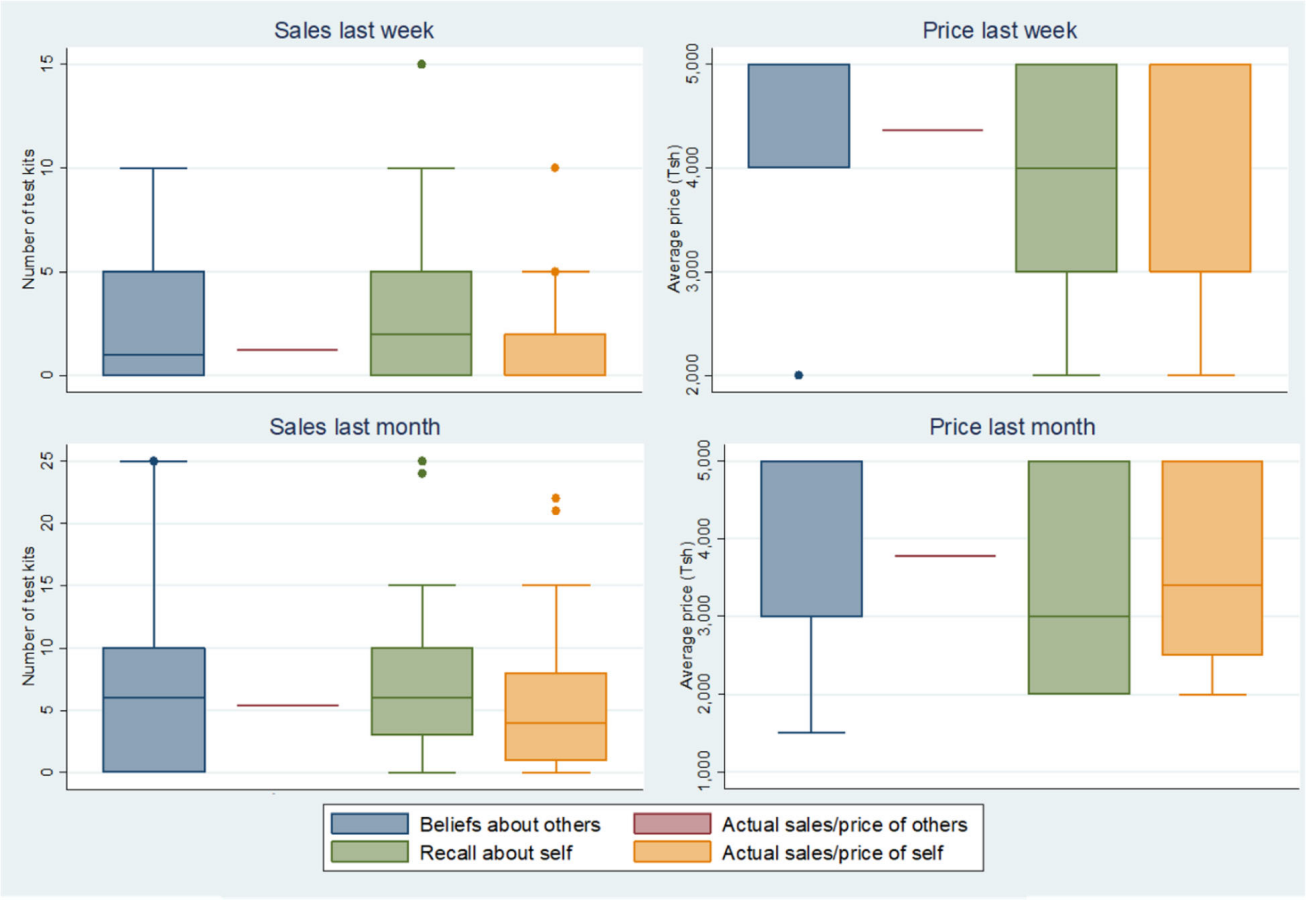
Accuracy of recall and beliefs about HIV self-test kit sales and pricing. *Box plot of shop owners’ beliefs about other shops’ sales volume and pricing of HIV self-test kits and recall of their own sales volume and pricing in the last week and month respectively, measured at the end of the study period (n = 25)*

Figure 2 shows how shopkeepers’ beliefs about other shops’ sales and pricing compared to recall of their own sales and pricing in the last week and month, respectively, measured at the end of the study. On average, shopkeepers were relatively accurate in estimating others’ sales volume, although a substantial minority severely overestimated as evidenced by the right skew. On average, shopkeepers underestimated others’ sales price. In contrast, shopkeepers were relatively accurate in recalling their owns sales and pricing.

**Fig. 3:**
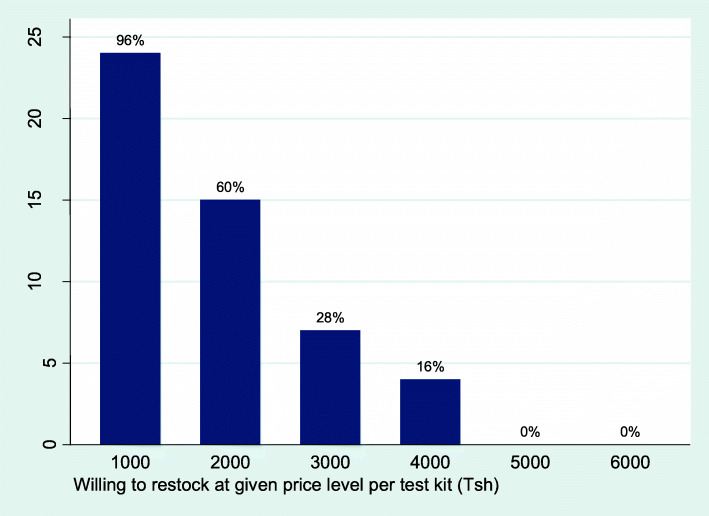
Willingness to restock HIV self-test kits at given price levels per kit (Tsh). *Note: $1 USD approx. 2300 Tsh. The subsidized global negotiated wholesale price is ~ 4600 Tsh per HIV self-test kit. Only 15/25 (60%) of shops were willing to restock at 2000 Tsh per test kit (the mean maximum willingness-to-pay to restock)*

Figure 3 shows the number of shops willing to restock at different price levels. Although 96% of shops were willing to restock at 1000 Tsh per self-test kit, this declined sharply as price levels increased and none were interested in restocking at 5000 Tsh or above. Willingness-to-pay to restock was positively associated with sales prices (both last month and predicted in the future) and volume last week, but was not associated with predicted sales volume or hypothetical demand for self-test kits at given price level increments in the future (Additional file 1: Table S4).

## Discussion

We collected some of the first systematic evidence on how drug shops in low-income countries set prices for new products like HIV self-test kits. Within our sample of shops in a semi-urban municipality in Tanzania, we found limited variation in observed sales prices over our 18-week study period. Given the novelty of the product and few reference points, sales prices on self-test kits varied (from 1000 to 6000 Tsh) across shops, but not within shops over time. This suggests that shopkeepers did not deliberately experiment with prices over time as would be expected if they were trying to maximize profits even though they were free to sell the kits at any price they wished. This observation contrasts with the typical profit-driven—even to the detriment of customers’ health needs—characterization of drug shops [24, 25], such as over-treating malaria regardless of diagnostic test results [26, 27]. We see little geospatial clustering of sales prices of self-test kits compared to prices of related existing products. At the end of the study, shopkeepers reported relatively inaccurate beliefs about other shops’ sales volume and prices, suggesting that shopkeepers might not have found it sufficiently worthwhile to investigate how much competitors were selling self-test kits for or the demand they were experiencing, even in a small localized urban hub (2 km squared). It is unclear whether local markets simply needed more time to converge on a market price for HIV self-test kits, or whether HIV self-test kits are different from other reproductive health products in ways we do not yet understand.

After having some time to learn about the product and consumer demand, shopkeepers’ interest in restocking HIV self-test kits at the end of the study was limited, even at highly subsidized prices. We add to the few studies estimating willingness-to-pay for HIV self-testing using more rigorous incentive-compatible revealed preference measures [19, 20], contributing the first evidence on willingness-to-pay on the supply-side (shopkeepers’ willingness to restock); previous studies have exclusively focused on estimating consumer demand (customers’ willingness to purchase). Only 60% of shops were willing to restock at 2000 Tsh per self-test kit (the mean maximum willingness-to-pay). Given that markups for related products were between 100 and 300% (Additional file 1: Fig. S4) and that the current subsidized global wholesale price for the oral HIV self-test kits (Orasure) is approximately 4600 Tsh ($2 USD) [28], HIV self-test kits are unlikely to be commercially viable in this setting without further demand creation and/or cost reduction. This finding stresses the importance of assessing shops’ interest in stocking and selling a given product in addition to consumer demand when considering private sector distribution strategies for HIV, sexual reproductive health, and other public health products.

What explains the apparent lack of variation in pricing and convergence over time? Qualitative data from the parent study provides several potential explanations [*Cabrera* et al.*, unpublished*], some of which resonate with previous findings in the literature. First, shopkeepers may be insufficiently motivated to experiment with prices due to low overall demand vis-à-vis low sales volumes. There were neither demand generation activities associated with the research study nor demand generation activities for HIV self-testing in this setting more generally. Qualitative evidence suggests that shopkeepers were more interested in stocking high turnover products, even with low profit margins, to maintain cash flow and attract customers to purchase other products. Second, shopkeepers discussed the time required to counsel customers on how to use the self-test kit, potentially jeopardizing sales to customers unwilling to wait [6]. Third, shops expressed some pro-social motivation for selling HIV self-test kits as community service, and may have been reluctant to increase prices at the cost of reducing availability [6, 29]. Future research into private sector distribution strategies should further investigate how drug shops make stocking and pricing decisions to better understand key factors that determine a product’s commercial viability and their trade-offs.

Lastly, we find limited evidence of price discrimination, contrary to evidence from many qualitative interviews with drug shopkeepers [6, 25]. Prices were lower for males and for 15–19 year olds compared to all other age groups, but these differences were smaller in magnitude compared to the price variation across shops. Although this suggests the lack of price gouging behavior, consistent with the lack of experimentation with prices overall, this also limits the potential for the general consumer market to cross-subsidize discounted products to specific vulnerable groups. The fact that we see differences in sales volume and prices by treatment arm for a complementary intervention promoting adolescent girls and young women friendly services suggests that interventions targeting specific groups and products through drug shops should consider potential spillovers on related products and populations. Sales volumes were lower and prices higher in the treatment arm, which is consistent with several explanations. Shops in the treatment arm were potentially less motivated to sell test kits, either from being preoccupied with the intervention or more perceived gain from the additional sales and customers that the intervention attracted. Alternatively, shops in the treatment arm may have saturated local demand, especially among AGYW, although we see lower sales across all age groups compared with shops in the control arm.

### Limitations

Our study has several limitations. First, sales and pricing behavior observed in our study may deviate from real-world settings. Self-test kits were given to shops for free, which may have influenced shopkeepers’ price expectations and accentuated their pro-sociality, but there is limited evidence of such anchoring from the literature [21, 30]. In fact, first distributing free samples when demand is uncertain is a common marketing strategy for product introduction. In this case, the future supply of HIV self-test kits was also uncertain. HIV self-test kits were approved for general sale at the end of the study and have yet to be distributed in the market or health system, so shops may have varied expectations of future supply. Second, we only measured age and sex of customers; we cannot rule out price discrimination along unobserved dimensions, which we believe to be unlikely given the low sales volume and limited variation in sales prices within shops overall. Third, our study is restricted by the small sample size, challenged further by low sales volumes per shop and differences in total sales and average prices by treatment arm in the parent study (although this is controlled for in all models). This finding underscores the inherent difficulty in measuring supply-side willingness-to-pay. Note that the existence and direction of treatment effects were ambiguous a priori – complementary interventions could have increased sales by attracting more customers overall. That shops in the treatment arm sold fewer self-test kits to the general population (lower sales volume, higher prices) is a valuable finding in of itself, with implications for program scale-up and implementation planning. Other than our parent study, we are unaware of other major interventions for HIV self-testing occurring concurrently in our study area (such as demand creation activities) that may have influenced our results. Furthermore, at the time of the study, HIV self-testing was not yet approved by the government outside of the research setting, so it is unlikely that other sources of HIV self-testing were available. Lastly, there is potential for measurement error with sales volume and pricing. We only measure final sales prices, which could differ from the initial price offered to the customer. Sales records were also self-reported although we tried to mitigate this by triangulating against stock audits and anonymous customer feedback forms. New tools that help automate and systematize inventory management can help drug shops better manage their operations, whilst improving data collection.

## Conclusion

There was limited variation in sales prices, low sales volume, and low demand among shopkeepers to restock HIV self-test kits at the end of the study. Given the subsidized global wholesale price ($2 USD or ~ 4600 Tsh), further demand creation and/or cost-reduction is needed before self-test kits can become commercially viable in this setting. Further research is needed to understand how shopkeepers make sales and pricing decisions when demand generation activities are present. Public health policymakers should be cautious when introducing a new product into the retail market given this experience with HIV self-testing, since there may be little willingness to experiment and stock when sellers are uncertain about demand. An integrated approach with demand creation is needed.

## Supplementary Information

**Additional file 1: Fig. S1.** Boxplot of sales volume per shop per week over the study period. **Table S1.** Shop characteristics and HIV self-test kit sales and pricing by intervention treatment arm. **Fig. S2.** Mean price of test kits sold per shop over the study period. **Fig. S3.** Geospatial clustering of related products vs HIVST. **Table S2.** Association between sales price and observable buyer characteristics (customer feedback forms). **Table S3.** Association between sales price, sales volume, and lagged sales volume. **Table S4.** Association between maximum willingness-to-pay to restock and sales volume and prices. **Fig. S4.** Profit and percentage market of related product. **Table S5.** Robustness of association between HIV self-test kit sales price and customer characteristics to shop/week fixed/random effects.

## Data Availability

The datasets used and/or analysed during the current study are available from the corresponding author on reasonable request.

## Abbreviations

ADDO: Accredited Drug Dispensing Outlets
AGYW: Adolescent girls and young women
SRH: Sexual and reproductive health
SD: Standard Deviation

## Footnotes

1 This refers to methods with real stakes where respondents’ best strategy is to answer truthfully, in contrast to hypothetical questions.

2 This represents 16% of all drug shops in Shinyanga Urban District (*N* = 163) at the time of the study. The 18-week study period was pre-determined based on implementation and data collection considerations for the parent study.

3 Sales were lower (188 vs. 326 self-test kits sold) and prices higher (median price of 4000 vs. 3000 Tsh) among shops in the treatment arm versus the control arm of the parent study (Additional file 1: Table S1). We control for treatment assignment in all regression models accordingly.

4 These are calculated by exponentiating the coefficients reported in Table 2 given our use of a log-linear model. For example, − 0.135 = e^(− 0.145)-1 (customer sex).
