# Supplementary material for: Sales and pricing decisions for HIV self-test kits among local drug shops in Tanzania: a prospective cohort study
Source: BMC Health Serv Res. 2021 May 6;21:434. doi: 10.1186/s12913-021-06432-1 (PMC8101213; doi:10.1186/s12913-021-06432-1)
Supplement: Supplementary file 1 — Additional file 1: Fig. S1. Boxplot of sales volume per shop per week over the study period. Table S1. Shop characteristics and HIV self-test kit sales and pricing by intervention treatment arm. Fig. S2. Mean price of test kits sold per shop over the study period. Fig. S3. Geospatial clustering of related products vs HIVST. Table S2. Association between sales price and observable buyer characteristics (customer feedback forms). Table S3. Association between sales price, sales volume, and lagged sales volume. Table S4. Association between maximum willingness-to-pay to restock and sales volume and prices. Fig. S4. Profit and percentage market of related product. Table S5. Robustness of association between HIV self-test kit sales price and customer characteristics to shop/week fixed/random effects. [file 12913_2021_6432_MOESM1_ESM.pdf]

Supplementary Information

Sales and pricing decisions for HIV self-test kits among local drug shops in Tanzania: a prospective cohort study

Calvin Chiu,[1§] MA, Lauren A. Hunter,[1] MPH, Sandra I. McCoy,[1] PhD MPH, Rashid Mfaume,[2] MD MPH, Prosper Njau,[3,4] MD MS, Jenny X. Liu,[5] PhD MPP

[1]School of Public Health; University of California, Berkeley; Berkeley, California, U.S.

[2]Shinyanga Regional Medical Office; Shinyanga, Tanzania.

[3]Health for a Prosperous Nation; Dar es Salaam, Tanzania.

[4]National AIDS Control Programme; Ministry of Health, Community Development, Gender, Elderly, and Children; Dar es Salaam, Tanzania.

[5]Institute for Health and Aging; Bixby Center for Global Reproductive Health; University of California, San Francisco; San Francisco, California, U.S.

[§] **Corresponding author**: Calvin Chiu

Address: 2121 Berkeley Way, Room 5302, Berkeley, CA 94720-7360

Phone Number: (+1) 510-292-1980

Email: calvin_chiu@berkeley.edu

Figure S1: Boxplot of sales volume per shop per week over the study period

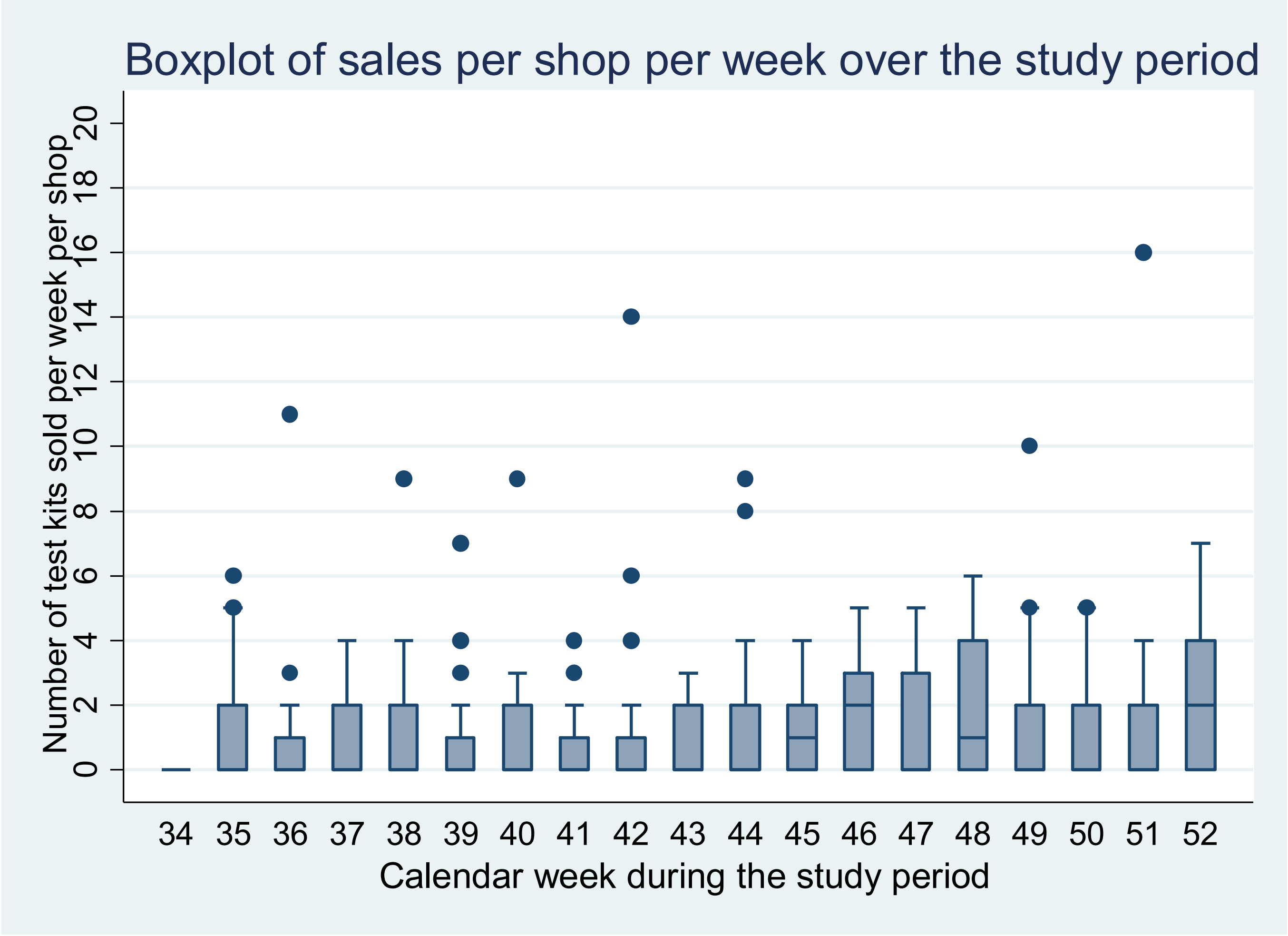

Each boxplot shows the distribution of number of test kits sold per week per shop over the study period. In most weeks, there is a long right tail and some outliers, indicating a distribution that is skewed to the right.

Table S1: Shop characteristics and HIV self-test kit sales and pricing by intervention treatment arm

	Control Arm			Treatment Arm			
	**n (%)**	**Median (IQR)**	**N**	**n (%)**	**Median (IQR)**	**N**	**P-Value**
Shop Characteristics (N=26)							
Location			13			13	1.000
- Urban	10 (77%)			10 (77%)			
- Peri-Urban	3 (23%)			3 (23%)			
Years in business			13			13	0.557
- 1 year or less	4 (31%)			2 (15%)			
- 1-5 years	1 (8%)			3 (23%)			
- 5-10 years	2 (15%)			1 (8%)			
- More than 10 years	6 (46%)			7 (54%)			
Number of employees (including owner)		1 (0)	13		1 (1)	13	1.000
HIV self-test kit sales (N=514)							
Over the study period	326 (63%)		514	188 (37%)		514	
Per week (18 weeks)		18 (10)	18		9 (8)	17	0.003**
Per shop (23 shops)		19 (26)	13		18 (22)	10	0.373
Per shop per week		0 (2)	247		0 (2)	190	0.094
Sex of customer			313			187	
- Female	80 (26%)			63 (34%)			0.052
- Male	233 (74%)			124 (66%)			
Age of customer			322			185	0.287
- 15-19	15 (5%)			3 (2%)			
- 20-24	50 (16%)			23 (12%)			
- 25-34	126 (39%)			79 (43%)			
- 35-44	97 (30%)			63 (34%)			
- 45+	34 (11%)			17 (9%)			
**Price per kit (Tsh) (1USD ~2,300 Tsh)**							
Over the study period		3000 (3000)	326		4000 (3000)	187	0.066
Number of test kits sold at							
- 1000 Tsh	36 (11%)			0 (0%)			
- 2000 Tsh	77 (24%)			56 (30%)			
- 2500 Tsh	11 (3%)			14 (7%)			
- 3000 Tsh	50 (15%)			17 (9%)			

- 4000 Tsh	21 (6%)			19 (10%)			
- 5000 Tsh	131 (40%)			80 (43%)			
- 6000 Tsh	0 (0%)			1 (1%)			
Mean price per shop (23 shops)		2567 (2842)	13		4083 (2083)	10	0.221
Median price per shop (23 shops)		3000 (3000)	13		4000 (2500)	10	0.374
By sex of customer							
- Male		3000 (3000)	233		4000 (3000)	124	0.268
- Female		3000 (3000)	80		4500 (3000)	62	0.160
By age of customer							
- 15-19		1000 (1000)	15		2500 (500)	3	0.016*
- 20-24		2250 (4000)	50		5000 (3000)	23	0.006**
- 25-34		3000 (3000)	126		3500 (3000)	78	0.511
- 35-44		4000 (3000)	97		4000 (3000)	63	0.789
- 45+		5000 (1000)	34		5000 (2000)	17	0.266
Price of existing related products (Tsh)							
Emergency contraception		5000 (2000)	7		5000 (0)	8	0.120
Oral contraception		1500 (0)	10		1625 (500)	10	0.160
Pregnancy test		1000 (0)	14		1000 (0)	11	0.975
**Beliefs about future demand (N=25)**							
Predicted sales next week if sold at							
- 0 Tsh (Free)		20 (14)	12		15 (10)	13	0.280
- 2,000 Tsh		15 (9.5)	12		8 (6)	13	0.427
- 5,000 Tsh		5 (6.25)	12		3 (3)	13	0.004**
- 10,000 Tsh		0.5 (3)	12		0 (0.0)	13	0.016*
Maximum willingness-to-pay to restock		2000 (1500)	12		1000 (1000)	13	0.500
Number of shops that restocked n(%)	3 (25%)		12	3 (23%)		13	0.915

1 From t-tests comparing differences in means by treatment arm for continuous variables and from chi-squared tests for categorical variables. * p<0.05, **p<0.01, ***p<0.001. Rows where N does not align with the denominator (E.g. Age of customer, sex of customer) for the level of observation (shop, test-kit, week, etc.) reflect observations where data from some variables is missing.

Figure S2: Mean price of test kits sold per shop over the study period

Each line represents the average price of test kits per week by a given shop over the study period. Shops chose different price levels initially and did not converge towards a market price over the study period.

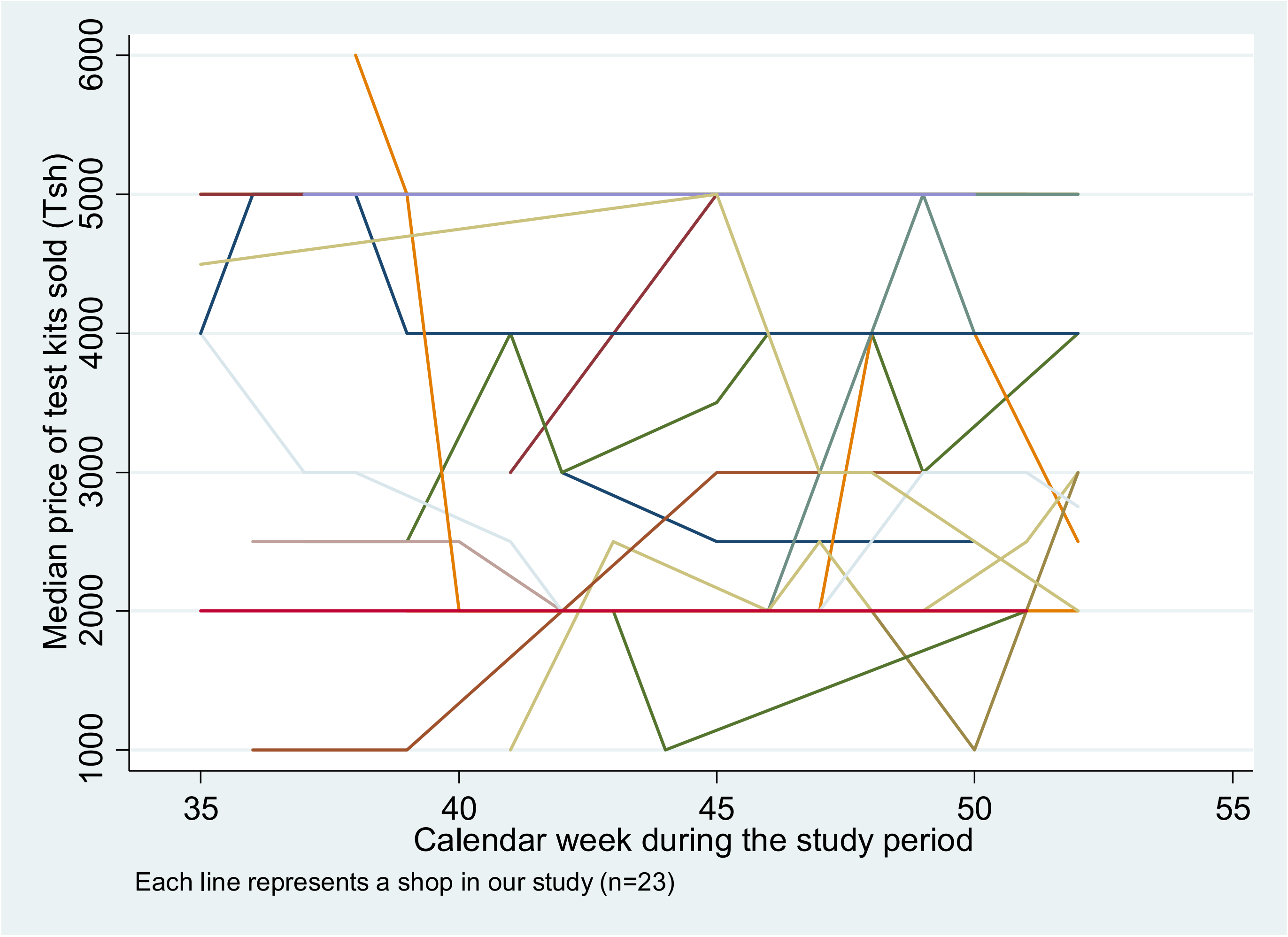

Figure S3: Geospatial clustering of related products vs HIVST

Each point represents the average price of a specific product sold by a given shop in our study. The prices of existing related products (emergency contraception, oral contraception, and pregnancy tests) cluster geospatially, whereas no such patterns exist for the price of HIVST or shop owners' beliefs about others' sales price. Coordinates of shops are jittered by adding random perturbations for confidentiality.

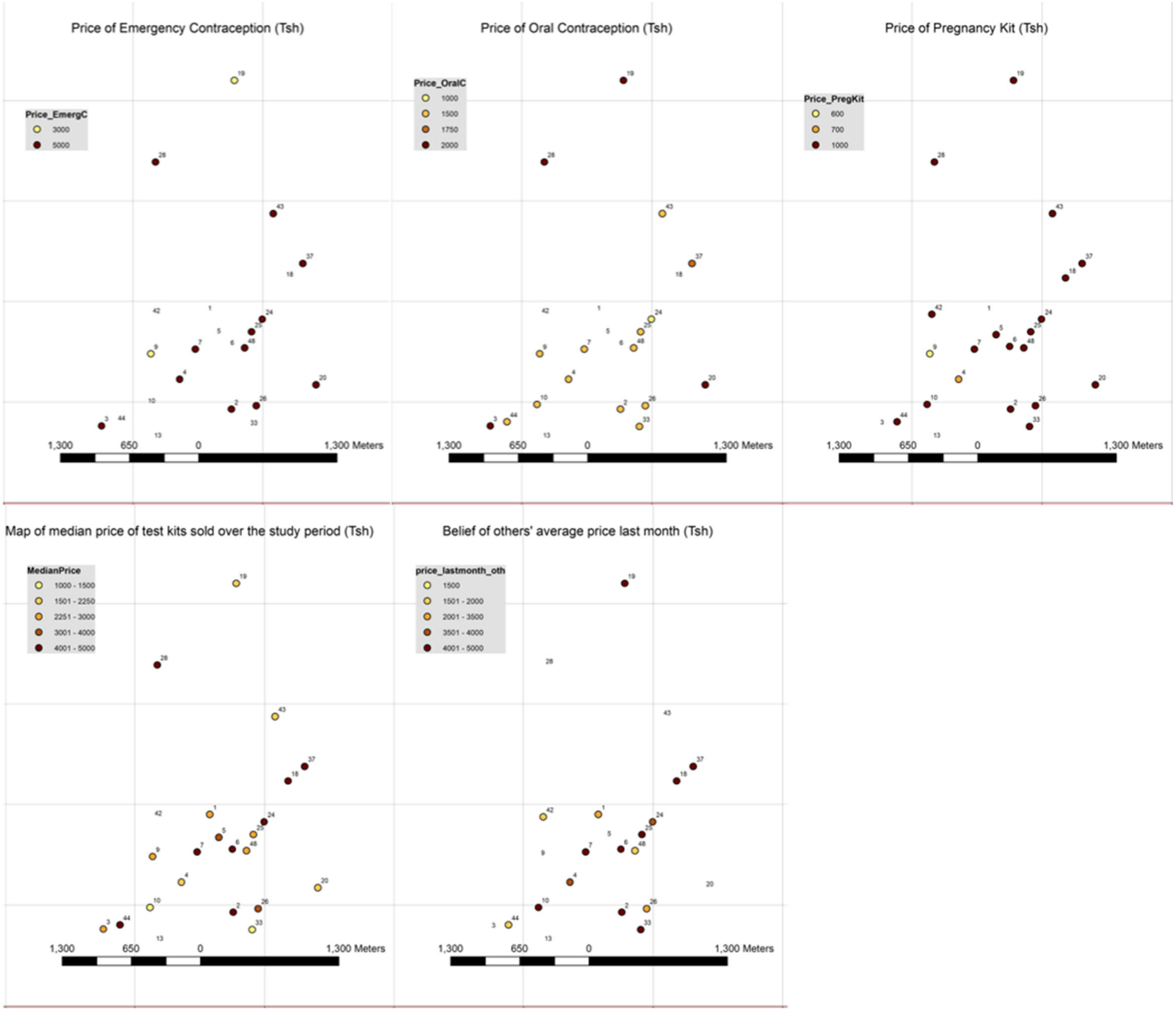

Table S2: Association between sales price and observable buyer characteristics (customer feedback forms)

**Linear regression on log price of test kits sold (Coefficient, standard error)**						
**Model**	**N (%)**	**Age**	**Sex**	**Age-Sex**	**All**	**All (age-sex)**
**Age**			-			
- 15-24	31 (11%)	ref	-	ref	ref	ref
- 25-34	123 (43%)	0.180 (0.867)	-	0.53** (0.152)	0.026 (0.044)	0.305 (0.192)
- 35-44	95 (33%)	0.436* (0.176)	-	0.548** (0.140)	0.052 (0.056)	0.254 (0.186)
- 45-54	35 (12%)	0.396 (0.198)	-	0.838*** (0.167)	0.048 (0.050)	0.328 (0.166)
**Sex** (Male)	184 (65%)	-	-0.113 (0.103)	0.240 (0.248)	-0.009 (0.016)	0.266 (0.181)
**P-values from F-tests of joint significance**						
Age		0.095	-	<0.001	0.731	0.147
Sex		-	0.287	0.346	0.607	0.159
Age x Sex		-	-	0.019	-	0.175
Shop		-	-	-	<0.001	<0.001
Week		-	-	-	<0.001	<0.001
Shop x Week		-	-	-	-	-
**Controls**						
- Treatment status		X	X	X	X	X
- Shop fixed effects		-	-	-	X	X
- Week fixed effects		-	-	-	X	X
N		218	218	216	216	216
* p<0.05, **p<0.01, ***p<0.001. Standard errors are clustered at the shop level unless specified otherwise (^). Price data from anonymous customer feedback forms (n=289; 284 have non-missing age and sex). Mean (SD): 3756 (1620); Median: 4000; Range: 1000-10000 (Tsh).						

Table S3: Association between sales price, sales volume, and lagged sales volume

**Linear regression on median price of test kits sold in a given week (Coefficient, standard error)**								
**Quantity sold**	-132.1*<br>(55.93)	-128.6*<br>(56.25)	-54.60<br>(37.57)	-133.2<br>(73.47)	-130.5*<br>(52.10)	-127.3*<br>(52.93)	-59.80<br>(39.18)	-131.3<br>(69.40)
Intercept	4123***<br>(305.6)	4044***<br>(440.8)	2748***<br>(84.52)	4696***<br>(361.4)	4158***<br>(327.4)	4079***<br>(460.8)	2811***<br>(116.8)	4690***<br>(355.2)
Lagged quantity sold	-	-	-	-	-24.81<br>(62.31)	-22.14<br>(62.42)	-51.78<br>(29.84)	-27.89<br>(65.09)
**Controls**								
- Treatment status	-	X	-	-	-	X	-	-
- Shop fixed effects	-	-	X	-	-	-	X	-
- Week fixed effects	-	-	-	X	-	-	-	X
N	176	176	176	176	176	176	176	176
* p<0.05, **p<0.01, ***p<0.001. Standard errors are clustered at the shop level								

Table S4: Association between maximum willingness-to-pay to restock and sales volume and prices

Linear regression on maximum willingness-to-pay to restock per test kit (Tsh) Coefficient (Standard error)											
	**Sales volume**						**Price**					
	**Demand at hypothetical prices**	**Last week**	**Last month**	**Over the study period**	**Next week**	**Next month**	**Last week**	**Last month**	**Over study period (Mean)**	**Over study period (Median)**	**Next week**	**Next month**
Intercept	2328** (651)	1730*** (248)	1927*** (318)	1614** (424)	1634** (443)	1540*** (391)	290 (1316)	307 (729)	763 (708)	886 (665)	-42.11 (570)	72.5 (605)
Coefficient		211.3* (96.78)	13.36 (39.26)	16.47 (14.53)	93.00 (95.96)	38.48 (26.68)	0.484 (0.321)	0.490* (0.190)	0.347 (0.187)	0.315 (0.175)	0.540** (0.149)	0.536** (0.154)
- At 0 Tsh	-40.20 (32.72)											
- At 2,000 Tsh	6.10 (57.43)											
- At 5,000 Tsh	-38.99 (168.3)											
- At 10,000 Tsh	375.4 (322.5)											
- Ascending order	679.1 (520.6)											
P-value	0.485	0.040	0.737	0.270	0.343	0.163	0.166	0.021	0.078	0.087	0.002	0.002

* p<0.05, **p<0.01, ***p<0.001. Standard errors in parenthesis

Figure S4: Profit and percentage market of related product

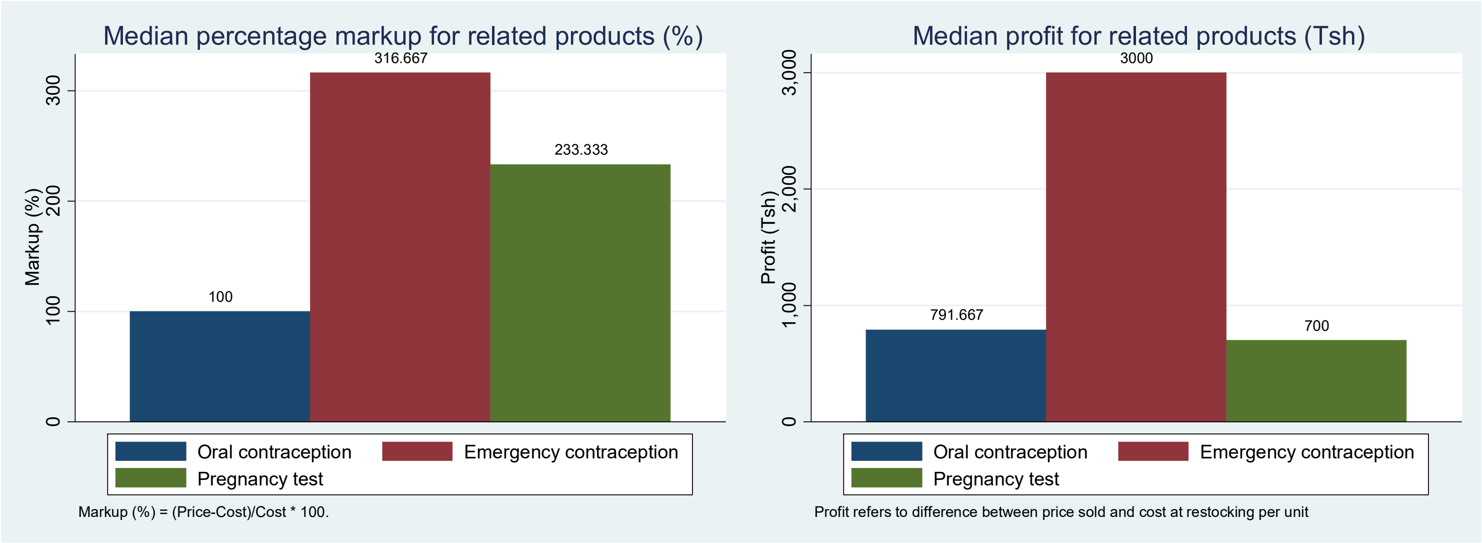

Table S5: Robustness of association between HIV self-test kit sales price and customer characteristics to shop/week fixed/random effects

Linear mixed models of log price of test kits sold (Coefficient, standard error)									
**N=492**	**(1)**	**(2)**	**(3)**	**(4)**	**(5)**	**(6)**	**(7)**	**(8)**	**(9)**
**Model**	**Naïve**	**Shop RE**	**Shop FE**	**Time RE†**	**Time FE†**	**Shop FE Time RE†**	**Shop RE Time FE**	**Shop RE Time RE**	**Shop FE Time FE**
**Shop**	-	RE	FE	-	-	FE	RE	RE	FE
**Time** (Week)	-	-	-	RE	FE	RE	FE	RE	FE
**Age**									
- 15-19	ref	ref	ref	ref	ref	ref	ref	ref	ref
- 20-24	0.367* (0.149)	0.011 (0.038)	-0.007 (0.037)	0.484*** (0.095)	0.637*** (0.096)	0.047 (0.060)	0.010* (0.044)	0.031 (0.030)	0.082 (0.044)
- 25-34	0.253 (0.132)	0.044 (0.029)	0.030 (0.028)	0.353*** (0.063)	0.481*** (0.053)	0.078 (0.047)	0.121*** (0.036)	0.017 (0.011)	0.107** (0.036)
- 35-44	0.338* (0.149)	0.014 (0.045)	-0.003 (0.044)	0.422** (0.091)	0.527*** (0.077)	0.041 (0.055)	0.081 (0.053)	0.012 (0.013)	0.065 (0.055)
- 45-54	0.535*** (0.155)	0.066*** (0.019)	0.045* (0.019)	0.639*** (0.120)	0.767*** (0.130)	0.092 (0.064)	0.139** (0.052)	0.014 (0.010)	0.120* (0.051)
**Sex** (Male)	-0.608** (0.200)	-0.206* (0.098)	-0.212* (0.097)	-0.489** (0.167)	-0.324* (0.155)	-0.171 (0.118)	-0.138* (0.057)	-0.007 (0.015)	-0.145** (0.056)
**P-values from F-tests of joint significance**									
Age	0.010	<0.001	0.007	<0.001	<0.001	0.345	0.001	0.043	0.005
Sex	0.002	0.035	0.029	0.003	0.037	0.147	0.016	0.655	0.010
Age x Sex	0.042	0.274	0.235	0.031	0.113	0.547	0.180	0.640	0.152
**P-values from Likelihood Ratio tests for model specification**									

- Model vs Naive	-	<0.001	<0.001	0.063	0.012	<0.001	<0.001	<0.001	<0.001
- Model vs Shop FE	-	-	-	-	-	0.002	-	-	<0.001
- Model vs Time FE	-	-	-	-	-	-	<0.001	-	<0.001
**P-values from Hausman tests for model specification**									
- FE vs RE	-	-	0.003	-	0.006	-	-	-	-

* p<0.05, **p<0.01, ***p<0.001. RE: Random Effects; FE: Fixed Effects. All models include treatment status, age, sex and age-sex interaction terms. Standard errors are clustered at the shop level unless specified otherwise (†). Only coefficients for customer age and sex are reported for brevity. P-values from Likelihood Ratio tests against the null hypothesis that the restricted model fits the data sufficiently. Rejecting the null hypothesis at conventional statistical significance levels leads us to reject the restricted model specification for the unrestricted model. P-values from Hausman tests against the null hypothesis that the random effects estimator is efficient and consistent. Rejecting the null hypothesis leads us to reject the random effects model for the fixed effects model.
